# Supplementary material for: Genetic Engineering of Lesquerella with Increased Ricinoleic Acid Content in Seed Oil
Source: Plants (Basel). 2021 May 29;10(6):1093. doi: 10.3390/plants10061093 (PMC8230273; doi:10.3390/plants10061093)
Supplement: Supplementary file 1 [file plants-10-01093-s001.zip › plants-1221941-supplementary.pdf]

Table S1. Estimated number of *transgene* locus for each transgenic line (T_1 seeds).

T_1 seeds	number of seedlings Basta^R	Basta^S	Estimated transgenic locus number
1	47	3	2
2	45	3	2
3	44	3	2
4	46	3	2
5	46	3	2
6	50	0	>2
7	36	11	1
8	47	3	2
9	47	3	2
10	37	12	1
11	45	3	2
12	46	3	2
13	36	12	1
14	47	3	2
15	38	11	1
16	35	10	1

Seeds were germinated on germination medium containing Basta at 1mg/l for 3 weeks. R, resistance seedlings showing normal developed with healthy cotyledons and 2-4 true leaves. S, sensitive seedlings showing arrested development with yellow cotyledons and no true leaf.

Table S2. Fatty acid composition (mole %) in T_1 seeds expressing *AtFAD3 RNAi + CsFAE1 RNAi*

Line	16:0	16:1	18:0	20:0	20:1
wild-type	1.7 ± 0.2	0.6 ± 0.1	1.5 ± 0.3	0.1 ± 0.0	0.7 ± 0.1
line 1	1.9 ± 0.2	0.3 ± 0.1	1.2 ± 0.3	0.2 ± 0.1	0.7 ± 0.1
line 2	2.2 ± 0.1	0.6 ± 0.1	1.7 ± 0.0	0.3 ± 0.0	0.5 ± 0.1
line 3	2.0 ± 0.1	0.5 ± 0.0	1.5 ± 0.1	0.2 ± 0.0	0.7 ± 0.1
line 4	2.0 ± 0.2	0.5 ± 0.0	1.2 ± 0.1	0.2 ± 0.0	0.8 ± 0.1
line 5	1.9 ± 0.1	0.7 ± 0.1	1.3 ± 0.0	0.2 ± 0.1	0.7 ± 0.1
line 6	1.8 ± 0.1	0.5 ± 0.1	1.2 ± 0.1	0.2 ± 0.0	0.5 ± 0.1
line 7	1.6 ± 0.1	0.5 ± 0.1	1.1 ± 0.0	0.2 ± 0.1	0.7 ± 0.1
line 8	1.8 ± 0.1	0.5 ± 0.1	1.4 ± 0.0	0.2 ± 0.1	0.8 ± 0.1
line 9	1.9 ± 0.1	0.6 ± 0.0	1.2 ± 0.1	0.2 ± 0.0	0.6 ± 0.1
line 10	1.8 ± 0.1	0.5 ± 0.1	1.6 ± 0.1	0.2 ± 0.1	0.8 ± 0.1
line 11	1.7 ± 0.1	0.5 ± 0.1	1.3 ± 0.1	0.1 ± 0.0	0.7 ± 0.1
line 12	1.8 ± 0.1	0.5 ± 0.1	1.4 ± 0.0	0.2 ± 0.1	0.8 ± 0.1
line 13	1.4 ± 0.2	0.4 ± 0.1	1.2 ± 0.1	0.1 ± 0.1	0.7 ± 0.1
line 14	1.6 ± 0.2	0.5 ± 0.1	1.4 ± 0.0	0.2 ± 0.1	0.7 ± 0.1
line 15	1.3 ± 0.2	0.5 ± 0.1	1.3 ± 0.1	0.1 ± 0.0	0.8 ± 0.1
line 16	1.5 ± 0.1	0.5 ± 0.1	1.3 ± 0.0	0.1 ± 0.1	0.6 ± 0.0
average of transgenics	1.7 ± 0.2	0.5 ± 0.1	1.3 ± 0.1	0.2 ± 0.0	0.1 ± 0.1

Three or four replicates of 30-seed sample were measured for wild-type and each transgenic line. All data are averages of measurements ±SD. Fatty acid legend: 16:0 ispalmitic, 16:1 is palmitoleic, 18:0 is stearic18:0, 20:0 is arachidic, and 20:1 is eicosenoic acids.

Table S3. Fatty acid composition (mole %) in T_1 seeds expressing *CsFAD2 RNAi* + *AtFAD3 RNAi* + *CsFAE1 RNAi*

Line	16:0	16:1	18:0	20:0	20:1
wild-type	1.6 ± 0.2	0.6 ± 0.1	1.3 ± 0.1	0.1 ± 0.0	0.6 ± 0.1
line 1	1.8 ± 0.0	0.6 ± 0.0	1.0 ± 0.0	0.1 ± 0.0	0.6 ± 0.0
line 2	1.8 ± 0.0	0.6 ± 0.0	0.9 ± 0.1	0.1 ± 0.0	0.5 ± 0.0
line 3	2.0 ± 0.0	0.8 ± 0.0	1.0 ± 0.1	0.2 ± 0.0	0.6 ± 0.1
line 4	1.7 ± 0.1	0.4 ± 0.1	1.0 ± 0.2	0.1 ± 0.0	0.6 ± 0.0
line 5	2.1 ± 0.1	1.0 ± 0.0	1.0 ± 0.0	0.2 ± 0.0	0.6 ± 0.0
line 6	1.8 ± 0.1	0.6 ± 0.1	0.9 ± 0.1	0.2 ± 0.0	0.6 ± 0.1
line 7	1.8 ± 0.1	0.5 ± 0.1	1.0 ± 0.1	0.1 ± 0.0	0.5 ± 0.0
line 8	1.8 ± 0.1	0.8 ± 0.1	0.8 ± 0.0	0.2 ± 0.0	0.6 ± 0.0
line 9	1.6 ± 0.1	0.5 ± 0.1	1.0 ± 0.0	0.1 ± 0.0	0.6 ± 0.0
line 10	1.7 ± 0.2	0.6 ± 0.1	1.0 ± 0.0	0.2 ± 0.0	0.6 ± 0.0
line 11	1.7 ± 0.1	0.6 ± 0.2	1.3 ± 0.1	0.2 ± 0.0	0.5 ± 0.0
line 12	1.6 ± 0.1	0.5 ± 0.0	1.0 ± 0.1	0.1 ± 0.0	0.6 ± 0.0
line 13	1.4 ± 0.1	0.6 ± 0.1	1.2 ± 0.0	0.2 ± 0.0	0.6 ± 0.0
line 14	1.6 ± 0.2	0.5 ± 0.1	1.4 ± 0.0	0.2 ± 0.1	0.7 ± 0.1
line 15	1.3 ± 0.1	0.5 ± 0.1	1.3 ± 0.0	0.1 ± 0.0	0.7 ± 0.0
average of transgenics	1.7 ± 0.2	0.6 ± 0.1	1.0 ± 0.1	0.1 ± 0.0	0.6 ± 0.0

Triplicates of 30-seed sample were measured for wild-type and each transgenic line. All data are averages of measurements ±SD. Fatty acid legend: 16:0 ispalmitic, 16:1 is palmitoleic, 18:0 is stearic18:0, 20:0 is arachidic, and 20:1 is eicosenoic acids.

Table S4. Correlations between the fatty acids among transgenic lines

FA	FA	T_1 *FAE1i+FAD3i*[a]	T_2 *FAE1i+FAD3*i[b]	T_1 *FAE1i+FAD3i+FAD2i*[c]
18:1	18:2	0.33	0.02	-0.38
18:1	18:3	-0.60**	-0.25	-0.19
18:1	18:1OH	0.93***	0.71***	0.15
18:1	20:1OH	-0.97***	-0.85***	-0.75***
18:1	20:2OH	-0.66**	-0.05	-0.19
18:1	tHFA	-0.98***	-0.82***	-0.98***
18:2	18:3	-0.92***	-0.83***	-0.67***
18:2	18:1OH	0.35	0.10	-0.03
18:2	20:1OH	-0.29	-0.15	0.33
18:2	20:2OH	-0.86***	-0.53***	-0.70**
18:2	tHFA	-0.28	-0.37**	0.38*
18:3	18:1OH	-0.58*	-0.33*	0.03
18:3	20:1OH	0.53*	0.29**	0.04
18:3	20:2OH	0.91***	0.45***	0.91***
18:3	tHFA	0.51*	0.27	0.17
18:1OH	20:1OH	-0.99***	-0.94***	-0.75**
18:1OH	20:2OH	-0.62**	0.06	0.11
18:1OH	tHFA	-0.90***	-0.49***	-0.12
20:1OH	20:2OH	0.60*	-0.09	0.00
20:1OH	tHFA	0.96***	0.75***	0.78***
20:2OH	tHFA	0.62*	0.22	0.19

tHFA, total HFA. Total number of transgenic lines (or sample size) were included in calculation for a, n = 16; for b, n = 47; and for c, n = 15. *, $p < 0.05$; **, $p < 0.01$; ***; $p < 0.001$.
